# Serial evaluation of the serum lactate level with the SOFA score to predict mortality in patients with sepsis

**DOI:** 10.1038/s41598-023-33227-7

**Published:** 2023-04-18

**Authors:** Heemoon Park, Jinwoo Lee, Dong Kyu Oh, Mi Hyeon Park, Chae-Man Lim, Sang-Min Lee, Hong Yeul Lee, the Korean Sepsis Alliance (KSA) Investigators

**Affiliations:** 1 Division of Pulmonary and Critical Care Medicine, Department of Internal Medicine, Seoul National University Hospital, Seoul National University College of Medicine, Seoul, Republic of Korea; 2 Department of Pulmonary and Critical Care Medicine, Asan Medical Center, University of Ulsan College of Medicine, Seoul, Republic of Korea; 3 Department of Critical Care Medicine, Seoul National University Hospital, 101 Daehak-ro, Jongno-gu, Seoul, 03080 Republic of Korea

**Keywords:** Medical research, Infectious diseases, Respiratory tract diseases

## Abstract

In patients with sepsis, outcome prediction plays an important role in influencing therapeutic decision making. In this nationwide, prospective, observational cohort study of sepsis patients conducted between September 2019 and December 2020, we evaluated a novel scoring system using serial Sequential Organ Failure Assessment (SOFA) scores and serum lactate to accurately predict mortality in sepsis. Based on the serum lactate score (Lac-score), patients were assigned to 5 categories: lactate < 2, ≥ 2 to < 4, ≥ 4 to < 8, ≥ 8 to < 12, and ≥ 12 mmol/L. Lac-SOFA score was defined as the sum of Lac-score and SOFA score. After screening 7113 patients, 379 were excluded and 6734 were included in analysis. In-hospital mortality AUROC for serial Lac-SOFA score from initial to ICU day 3 was significantly higher than that for serial SOFA score (initial, 0.679 vs. 0.656, day 1, 0.723 vs. 0.709, day 2, 0.760 vs. 0.747, and day 3, 0.797 vs. 0.781; DeLong's test, *p* < 0.001). The initial Lac-SOFA score significantly correlated with in-hospital mortality when the patients were divided into five classes based on 5-point intervals (*p* < 0.05). Serial evaluation of lactate levels with the SOFA score may improve the predictive accuracy of the SOFA score for determining mortality risk in sepsis patients.

## Introduction

The Sequential Organ Failure Assessment (SOFA) score is widely used to predict the prognosis of critically ill patients with sepsis^1–4^. Lactate levels are a reliable marker of sepsis-induced tissue hypoperfusion, which is correlated with mortality^5^. Lactate levels greater than 2 mmol/L (18 mg/dL) were used to define septic shock in a recently revised sepsis guideline^3^, and several studies have shown that lactate levels greater than 4 mmol/L and lactate clearance in sepsis is associated with mortality^6–10^. Until recently, there has been no study in which lactate levels were scored and evaluated in combination with existing scoring systems such as the SOFA score, Acute Physiology and Chronic Health Evaluation (APACHE) score, and Simplified Acute Physiology Score (SAPS) score. Recently, it has been suggested that the SOFA score, which has been used for 25 years, needs to be updated^11^. As the blood lactate level is easily monitored and is strongly associated with mortality and morbidity in critically ill patients, it could be considered as a new variable in an updated SOFA score^11^.

However, the calculations in existing scoring systems used for mortality prediction, including the APACHE and SAPS, use data obtained within the first 24 h, and do not consider changes in the patient due to the treatment following intensive care unit (ICU) admission^1^. Accordingly, there have been studies using the SOFA score or the APACHE score over time^12–14^. In addition, several studies have shown that changes in SOFA reflect the degree of organ dysfunction over time^1,15,16^. Ferreira et al.^1^ reported peak and total amount of organ dysfunction can be inferred through using mean SOFA score and highest SOFA score. In the management of critically ill patients with sepsis, outcome prediction plays an important role by influencing therapeutic decision-making, guiding resource allocation, and facilitating information-sharing or counseling of patients and their relatives by physicians^1,17,18^. However, existing scoring systems have primarily focused on a broad subset of critically ill patients and studies specific to patients with sepsis are lacking. In view of the limitations of existing scoring systems and the lack of studies focusing specifically on patients with sepsis, a specialized and optimal scoring system is needed to accurately predict the risk of death in patients with sepsis.

We hypothesized that serial evaluation of lactate levels with the SOFA score might improve the utility of the SOFA score for predicting in-hospital mortality in patients with sepsis. We evaluated a novel scoring system that used serial SOFA scores and serum lactate levels to predict mortality in patients with sepsis.

## Definition

Initial Lac-score was defined as the serum lactate level on time zero of sepsis, and indicated five categories: 0 (lactate < 2 mmol/L), 1 (2 mmol/L ≤ lactate < 4 mmol/L), 2 (4 mmol/L ≤ lactate < 8 mmol/L), 3 (8 mmol/L ≤ lactate < 12 mmol/L), and 4 (lactate ≥ 12 mmol/L). Initial Lac-SOFA score (range, 0–28) was defined as the sum of initial Lac-score and initial SOFA score (range, 0–24) on time zero of sepsis. Similarly, Lac-score ICU day-1, Lac-score ICU day-2, Lac-score ICU day-3, Lac-SOFA ICU day-1, Lac-SOFA ICU day-2, and Lac-SOFA ICU day-3 were derived. The Delta Lac-score was defined as the ratio of serum lactate on ICU day 3 to serum lactate on ICU day 1, and indicated four categories: 0 (ratio ≤ 0.5), 1 (0.5 < ratio ≤ 1), 2 (1 < ratio ≤ 1.5), and 3 (ratio > 1.5). Delta Lac-SOFA (range, 0–31) was defined as the sum of the Lac-SOFA ICU day-3 and Delta Lac-score (Table 1).

**Table 1:** Sequential Organ Failure Assessment (SOFA) score with the Lac-score and Delta Lac-score.

Variable	Score				
	0	1	2	3	4
Respiratory system					
PaO_2_/FiO_2_ (mmHg)	≥ 400	< 400	< 300	< 200 with respiratory support	< 100 with respiratory support
Hepatic system					
Bilirubin (mg/dL)	< 1.2	1.2–1.9	2.0–5.9	6.0–11.9	> 12.0
Cardiovascular system	MAP ≥ 70 mmHg	MAP < 70 mmHg	Dop < 5 or dob (any dose)^a^	Dop 5.1–15 or epi ≤ 0.1 or norepi ≤ 0.1^a^	Dop > 15 or epi > 0.1 or norepi > 0.1^a^
Coagulation					
Platelets × 10^3^/µL	≥ 150	< 150	< 100	< 50	< 20
Central nervous system					
Glasgow coma scale	15	13–14	10–12	6–9	< 6
Renal system					
Creatinine (mg/dL)	< 1.2	1.2–1.9	2.0–3.4	3.5–4.9	> 5.0
Urine output (mL/d)				< 500	< 200
Hypoperfusion status					
(Lac-score)					
Serum lactate (mmol/L)	< 2	≥ 2 and < 4	≥ 4 and < 8	≥ 8 and < 12	≥ 12
Progression of hypoperfusion					
(Delta Lac-score)					
The ratio of serum lactate on ICU day 3 to serum lactate on ICU day 1	≤ 0.5	> 0.5 and ≤ 1	> 1 and ≤ 1.5	> 1.5	

^a^Adrenergic agents administered for at least 1 h, all catecholamine doses represent µg/kg/min; Lac-SOFA score was defined as sum of Lac-score and SOFA score. Delta Lac-SOFA was defined as sum of Lac-SOFA ICU day-3 and Delta Lac-score.

*ICU* intensive care unit, *PaO*_*2*_ partial pressure of oxygen, *FiO*_*2*_ fraction of inspired oxygen, *MAP* mean arterial pressure, *Dop* dopamine, *Dob* dobutamine, *Epi* epinephrine, *Norepi* norepinephrine.

To evaluate and verify the predictive accuracy of the novel scoring system based on serial SOFA scores and serum lactate levels, two or more groups of patients with sepsis with different characteristics were needed: “non-ICU group” consisted of patients not admitted to the ICU; “early-ICU group” comprised patients admitted to the ICU within 6 h from time zero; and “delayed-ICU group” included patients admitted to the ICU 6 h after time zero^19^. The new scoring system was derived from the SOFA scores and serum lactate levels until day 3 of ICU admission.

## Results

Of the total 7,113 patients with sepsis registered in the sepsis registry between September 2019 and December 2020, 2 patients with initial SOFA score of 1 and 377 patients without initial serum lactate results and without available serum lactate results on day 1 of ICU were excluded. Patients were divided into non-ICU group (n = 3772), early-ICU group (n = 1187), and delayed-ICU group (n = 1775) (Supplementary Fig. S1 online).

### Process and results of imputation

#### Early-ICU group

In the early-ICU group, 19 patients did not have serum lactate values for ICU day 1. Among them, 13 patients who did not have serum lactate values on ICU day 2 or 3 were excluded. Of the remaining 6 patients, 4 patients had an initial lactate value that was tested within 2 h from time zero of sepsis; thus, the serum lactate level on ICU day 1 was replaced by the initial lactate level, and imputation was done with the other 2 patients. Thus, Lac-SOFA ICU day-1 was analyzed with 1,174 patients. One-hundred fifty patients did not have serum lactate values for ICU day 2. Among them, 139 patients who did not have available serum lactate values for ICU day 1 or 3 were excluded. Of the remaining 11 patients, one patient had an initial lactate value that was tested within 2 h from time zero of sepsis; thus, the serum lactate level on ICU day 1 was replaced by the initial lactate level, and imputation was done with these 11 patients. Thus, Lac-SOFA ICU day-2 was analyzed with 1048 patients. For ICU day 3, 329 patients did not have serum lactate values. Among them, 144 patients who did not have available serum lactate values on ICU day 1 or 2 were excluded. Of the remaining 185 patients, 2 patients had an initial lactate value that was tested within 2 h from time zero of sepsis; thus, the serum lactate level on ICU day 1 was replaced by the initial lactate level, and imputation was done with these 185 patients. Moreover, 135 patients did not have a SOFA score on ICU day 3; however, an imputation was performed based on the SOFA scores obtained for ICU days 1 and 2. After imputation, 12 of the SOFA scores calculated for ICU day 3 were negative and were replaced by zero. The serum lactate level calculated for ICU day 3 was negative and was replaced by zero in 33 patients. Thus, Lac-SOFA ICU day-3 was analyzed with 1043 patients.

Of the 1043 patients used for Lac-SOFA ICU day-3 analysis, 4 patients did not have serum lactate values for ICU day 1. Among them, one patient who did not have serum lactate values on ICU day 2 was excluded. Of the remaining 3 patients, one patient had an initial lactate value that was tested within 2 h from time zero of sepsis; thus, the serum lactate level on ICU day 1 was replaced by the initial lactate level, and imputation was done with the 2 patients. Because the serum lactate values on ICU days 1 or 3 were given as ranges, 4 patients who could not calculate Delta Lac-SOFA were excluded. Thus, Delta Lac-SOFA was analyzed with 1038 patients.

#### Delayed-ICU group

In the delayed-ICU group, 87 patients did not have serum lactate values for ICU day 1. Among them, 64 patients who did not have serum lactate values on ICU day 2 or 3 were excluded; imputation was done with 23 patients. Thus, Lac-SOFA ICU day-1 was analyzed with 1711 patients. Two-hundred seventy patients did not have serum lactate values for ICU day 2. Among them, 237 patients who did not have available serum lactate values on ICU day 1 or 3 were excluded, and imputation was done with 33 patients. Thus, Lac-SOFA ICU day-2 was analyzed with 1538 patients. Five hundred patients did not have serum lactate values for ICU day 3. Among them, 239 patients who did not have available serum lactate values on ICU day 1 or 2 were excluded, and imputation was done with these 261 patients. Moreover, 167 patients did not have SOFA score on ICU day 3; however, an imputation was performed based on the SOFA scores obtained for ICU days 1 and 2. After imputation, 15 of the SOFA scores calculated for ICU day 3 were negative and were replaced by zero. The calculated serum lactate level on ICU day 3 in 52 patients was negative and was replaced by zero. Thus, Lac-SOFA ICU day-3 was analyzed with 1536 patients.

Of the 1536 patients used for Lac-SOFA ICU day-3 analysis, 33 patients did not have serum lactate values for ICU day 1. Among them, 10 patients who did not have serum lactate values on ICU day 2 was excluded, and imputation was done with 23 patients. Because the serum lactate values for ICU day 1 or 3 were given as ranges, one patient was excluded as Delta Lac-SOFA could not be calculated. Thus, Delta Lac-SOFA was analyzed with 1525 patients.

### Baseline characteristics

The baseline characteristics of the entire study population were as follows: median age 73 (range, 19–104) years, male sex 58.8%, body mass index (BMI) 21.9 ± 0.1 kg/m^2^, serum lactate 3.7 ± 0.0 mmol/L, and initial SOFA score 6.3 ± 0.0. The baseline characteristics of the non-ICU, early-ICU, and delayed-ICU groups showed significant intergroup difference, except for the proportion of male sex and history of diseases, which means that these groups had different characteristics and were suitable for verifying the novel scoring system (Table 2).

**Table 2:** Baseline characteristics in the non-ICU, early-ICU, and delayed-ICU groups.

Variable	Non-ICU group (n = 3772)	Early-ICU group (n = 1187)	Delayed-ICU group (n = 1775)	*p*-value^d^
Age (years)^a^	73 (20–101)	72 (20–99)	73 (19–104)	0.007
Sex, male, n (%)^b^	2182 (58.1)	714 (59.5)	1062 (59.8)	0.401
BMI (kg/m^2^)^c^	21.5 ± 0.1	22.4 ± 0.1	22.3 ± 0.1	< 0.001
SOFA score, initial^c^	5.7 ± 0.1	7.6 ± 0.1	6.7 ± 0.1	< 0.001
Serum lactate level, initial^c^	3.3 ± 0.1	4.7 ± 0.1	3.9 ± 0.1	< 0.001
Underlying diseases, n (%)^b^				
Diabetes mellitus	1263 (33.6)	469 (39.1)	698 (39.3)	< 0.001
Chronic heart failure	278 (7.4)	78 (6.5)	131 (7.4)	0.553
Myocardial infarction	265 (7.1)	106 (8.8)	223 (12.6)	< 0.001
Chronic neurological disease	928 (24.7)	265 (22.1)	427 (24.1)	0.179
Chronic lung disease	635 (16.9)	146 (12.2)	290 (16.3)	< 0.001
Chronic kidney disease	440 (11.7)	195 (16.2)	299 (16.8)	< 0.001
Chronic liver disease	353 (9.4)	116 (9.7)	205 (11.5)	0.040
Hematologic malignancy	259 (6.9)	96 (8.0)	104 (5.9)	0.074
Solid malignancy	1567 (41.7)	345 (28.7)	498 (28.1)	< 0.001
Laboratory variables^c^				
White-cell count, 10^3^/μL	12.7 ± 0.2	13.0 ± 0.4	12.6 ± 0.2	0.613
Hemoglobin, g/dL	10.7 ± 0.0	10.4 ± 0.1	10.9 ± 0.1	< 0.001
Platelet count, 10^3^/μL	186 ± 1.9	170 ± 3.3	177 ± 2.8	< 0.001
Bilirubin level, mg/dL	1.8 ± 0.1	2.0 ± 0.1	1.8 ± 0.1	0.335
Blood urea nitrogen, mg/dL	34.3 ± 0.4	41.1 ± 0.9	39.8 ± 0.7	< 0.001
Creatinine level, mg/dL	1.6 ± 0.0	2.2 ± 0.1	2.1 ± 0.0	< 0.001
Lactate level, mmol/L	3.3 ± 0.0	4.7 ± 0.1	3.9 ± 0.1	< 0.001
Albumin, g/dL	2.9 ± 0.0	2.8 ± 0.0	2.9 ± 0.0	0.007
Procalcitonin, ng/mL	14.0 ± 0.8	25.4 ± 1.7	23.8 ± 1.6	< 0.001
Physiologic variables^c^				
Mean blood pressure, mmHg	74 ± 0	71 ± 1	72 ± 1	< 0.001
Heart rate, beats/min	107 ± 0	107 ± 1	106 ± 1	0.818
Respiratory rate, breaths/min	24 ± 0	25 ± 0	24 ± 0	< 0.001
Body temperature, °C	37.4 ± 0.0	37.2 ± 0.0	37.3 ± 0.0	< 0.001

^a^Values are presented as the median (range); ^b^Values are presented as number of subjects (percentage); ^c^Values are presented as the mean ± SE; ^d^Differences in three groups were tested with two-tailed Kruskal–Wallis test for continuous variables and two-tailed chi-squared test for dichotomous variables. *BMI* body mass index, *ICU* intensive care unit, *SOFA* Sequential Organ Failure Assessment score, *Lac-SOFA score* sum of SOFA score and Lac-score, *SE* standard error.

### Non-ICU group

Among non-ICU patients with sepsis, the area under the receiver-operating characteristic (ROC) curve (AUROC) of the in-hospital mortality for initial Lac-SOFA score was significantly higher than that of the initial SOFA score (0.694 ± 0.010 vs. 0.667 ± 0.011; p < 0.001) (Supplementary Fig. S2 online, Table 3). The initial Lac-score-based in-hospital mortality increased significantly from 0 to 4 points (p < 0.05) (Supplementary Table S1 online).

**Table 3:** AUROCs for in-hospital mortality based on the SOFA, Lac-SOFA, and Delta Lac-SOFA scores.

Variable	Non-ICU group		Early-ICU group		Delayed-ICU group	
	AUROC	*p*-value	AUROC	*p*-value	AUROC	*p*-value
SOFA, initial	0.667 ± 0.011	< 0.001	0.655 ± 0.017	< 0.001	0.598 ± 0.015	< 0.001
Lac-SOFA, initial	0.694 ± 0.010	< 0.001	0.678 ± 0.016	< 0.001	0.615 ± 0.015	< 0.001
SOFA, initial vs Lac-SOFA, initial		< 0.001^a^		< 0.001^a^		< 0.001^a^
SOFA on ICU day 1			0.710 ± 0.016	< 0.001	0.707 ± 0.013	< 0.001
Lac-SOFA ICU day-1			0.728 ± 0.016	< 0.001	0.718 ± 0.013	< 0.001
SOFA on ICU day 1 vs Lac-SOFA ICU day-1				< 0.001^a^		0.002^a^
SOFA on ICU day 2			0.757 ± 0.016	< 0.001	0.740 ± 0.014	< 0.001
Lac-SOFA ICU day-2			0.778 ± 0.015	< 0.001	0.749 ± 0.014	< 0.001
SOFA on ICU day 2 vs Lac-SOFA ICU day-2				< 0.001^a^		0.002^a^
SOFA on ICU day 3			0.789 ± 0.015	< 0.001	0.775 ± 0.013	< 0.001
Lac-SOFA ICU day-3			0.811 ± 0.014	< 0.001	0.787 ± 0.013	< 0.001
Delta Lac-SOFA			0.821 ± 0.014	< 0.001	0.793 ± 0.013	< 0.001
SOFA on ICU day 3 vs Lac-SOFA ICU day-3				< 0.001^a^		< 0.001^a^
Lac-SOFA ICU day-3 vs Delta Lac-SOFA				< 0.001^a^		0.002^a^

*ROC* receiver operating characteristic, *AUROC* area under the ROC curve, *ICU* intensive care unit, *SOFA* Sequential Organ Failure Assessment score, *Lac-SOFA score* sum of SOFA score and Lac-score, *Delta Lac-SOFA score* sum of Lac-SOFA ICU day-3 score and Delta Lac-score. ^a^Two-tailed DeLong's test for two correlated ROC curves.

### Early-ICU and delayed-ICU groups

The in-hospital mortality AUROC for serial Lac-SOFA scores was significantly higher than that of serial SOFA score in the early-ICU (initial, 0.678 ± 0.016 vs. 0.655 ± 0.017, ICU day 1, 0.728 ± 0.016 vs. 0.710 ± 0.016, ICU day 2, 0.778 ± 0.015 vs. 0.757 ± 0.016, ICU day 3, 0.811 ± 0.014 vs. 0.789 ± 0.015; p < 0.001) and delayed-ICU (initial, 0.615 ± 0.015 vs. 0.598 ± 0.015; p < 0.001, ICU day 1, 0.718 ± 0.013 vs. 0.707 ± 0.013, ICU day 2, 0.749 ± 0.014 vs. 0.740 ± 0.014, ICU day 3, 0.787 ± 0.013 vs. 0.775 ± 0.013, p = 0.002) groups (Table 3, Supplementary Fig. S2 online, Supplementary Fig. S3 online). When comparing Lac-SOFA ICU day-3 score and Delta Lac-SOFA score, the in-hospital mortality AUROC for the Delta Lac-SOFA score were significantly higher than that of the Lac-SOFA ICU day-3 score in the early-ICU (0.821 ± 0.014 vs. 0.811 ± 0.014; p < 0.001) and delayed-ICU (0.793 ± 0.013 vs. 0.787 ± 0.013; p < 0.001) groups (Table 3, Supplementary Fig. S4 online). The SOFA score on ICU day 3 and the serum lactate level on ICU day 3 had the highest in-hospital mortality AUROCs (Supplementary Table S2 online). The Lac-score from initial to ICU day 3-based in-hospital mortality increased significantly from 0 to 4 points (p < 0.05). Lac-score from ICU day 3 plus Delta Lac-score-based in-hospital mortality also increased significantly from 0 to 7 points (p < 0.05) (Supplementary Table S1 online). The Lac-score on ICU day 3 had the highest AUROC, and the Lac-score ICU day-3 plus Delta Lac-score AUROC was higher than that of the Lac-score ICU day-3 (Supplementary Table S2 online).

### Entire study population

There was no significant difference in the compliance with the hour-1 sepsis bundle according to the class of the Delta Lac-SOFA score (Supplementary Table S3 online). The in-hospital mortality increased as the score of each component of the SOFA score and Lac-score increased (Supplementary Fig. S5 online). The in-hospital mortality AUROC for the initial Lac-SOFA score was significantly higher than that of the initial SOFA score (0.679 ± 0.007 vs. 0.656 ± 0.008; p < 0.001). The in-hospital mortality AUROC for the Delta Lac-SOFA score (0.804 ± 0.010) was significantly higher than those of the Lac-SOFA (0.795 ± 0.010) and SOFA (0.779 ± 0.010) scores on ICU day 3 (p < 0.001). The in-hospital mortality AUROCs for the Lac-score on ICU day 3 and sum of the Lac-score on ICU day 3 and Delta Lac-score was lower than those of the SOFA and serum lactate level on ICU day 3. However, the in-hospital mortality AUROCs for the Lac-SOFA on ICU day 3 and Delta Lac-SOFA scores were significantly higher than those of the SOFA and serum lactate level on ICU day 3 (Table 3 and Supplementary Table S4). The in-hospital mortality of patients with an initial SOFA score ≤ 10 (n = 6050) was less than 43.1%. However, among these patients, the in-hospital mortality of patients with an initial Lac-SOFA score of 12, 13, or 14 was 52.8%, 58.3%, and 75.0%, respectively (Supplementary Table S5 online). Even in patients with the same initial SOFA score, the in-hospital mortality increased as the initial Lac-SOFA score increased (Supplementary Fig. S6 online).

**Figure 1:**
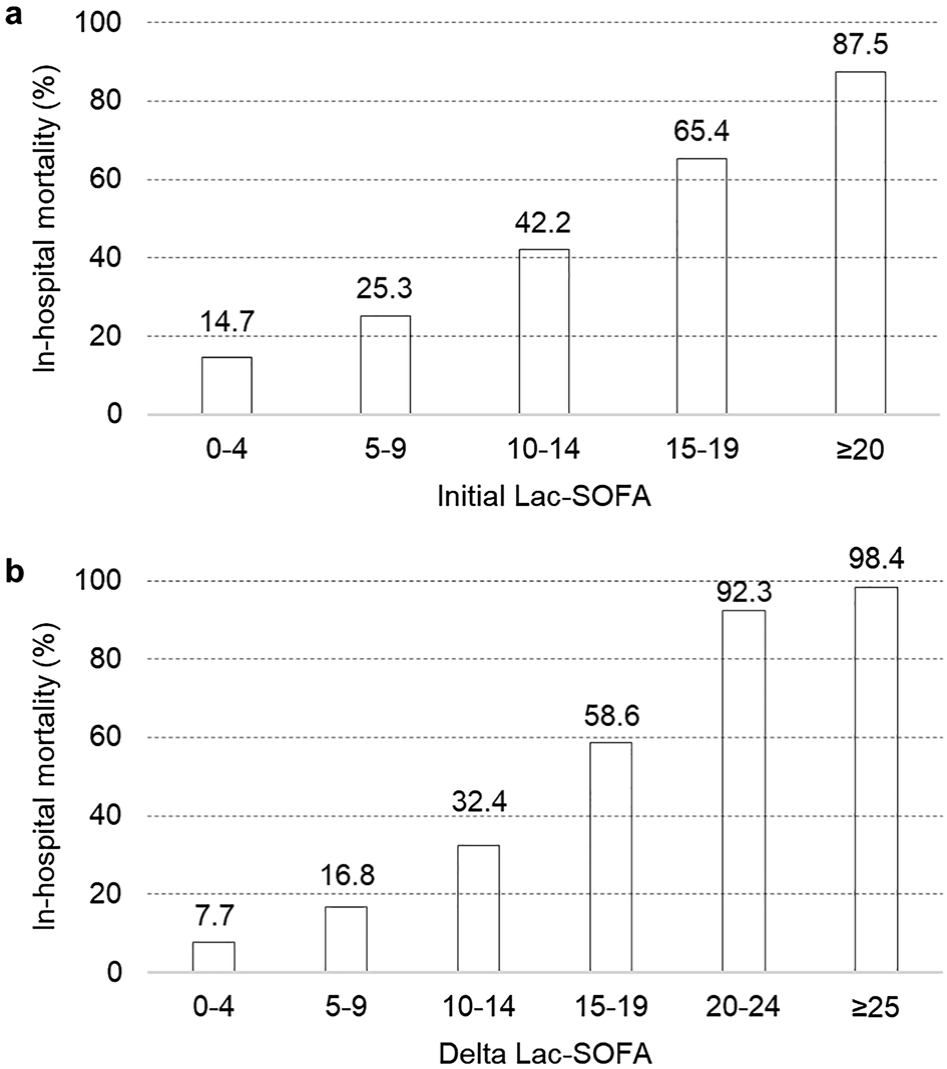
In-hospital mortality according to the changes in the initial Lac-SOFA score and Delta Lac-SOFA score. Patients were divided into five or six subgroups based on 5-point intervals of the (**a**) initial Lac-SOFA or (**b**) Delta Lac-SOFA scores. The numbers above the bar show the in-hospital mortality rate. *ICU* intensive care unit, *SOFA* Sequential Organ Failure Assessment score, *Lac-SOFA* sum of the SOFA score and the Lac-score, *Delta Lac-SOFA* sum of the Lac-SOFA ICU day-3 and Delta Lac-scores.

The initial Lac-SOFA and Delta Lac-SOFA scores significantly correlated with in-hospital mortality when the entire study population was divided into five or six subgroups based on 5-point intervals of the initial Lac-SOFA or Delta Lac-SOFA scores (p < 0.05); scores between 5 and 9 predicted mortality rates of 25.3% in the initial Lac-SOFA score and 16.8% in the Delta Lac-SOFA score, respectively. Moreover, an initial Lac-SOFA of ≥ 20 and Delta Lac-SOFA score of ≥ 25 predicted a mortality rate of 87.5% and 98.4%, respectively (Fig. 1).

**Figure 2:**
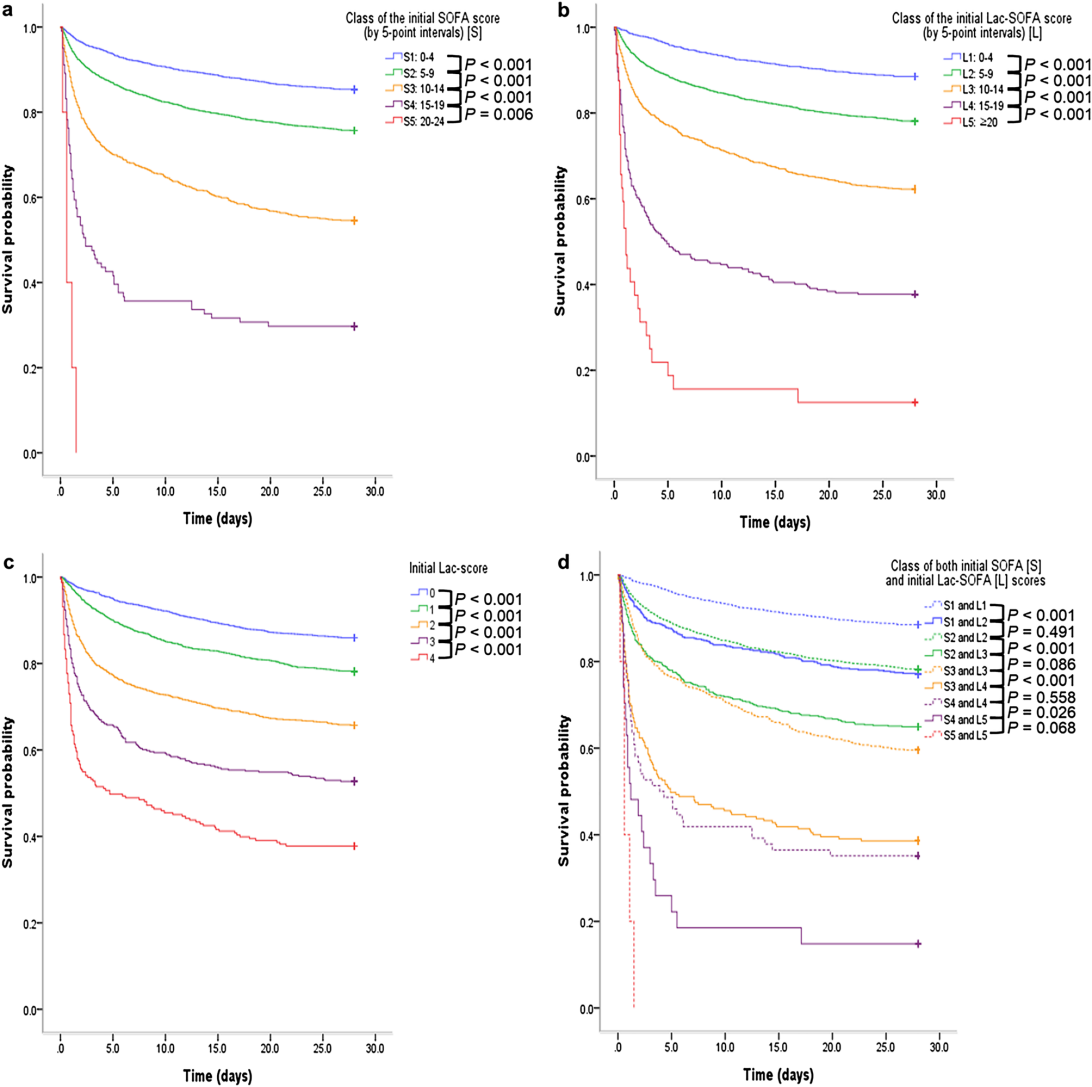
The survival probability from inclusion to day 28 according to initial SOFA, Lac-SOFA, and Lac-scores. Kaplan–Meier plots according to (**a**) class of the initial SOFA score (by 5-point intervals) [S], (**b**) class of the initial Lac-SOFA score (by 5-point intervals) [L], (**c**) initial Lac-score, (**d**) class of both initial SOFA [S] and initial Lac-SOFA [L] scores. The survival differences were analyzed using the two-tailed log-rank test. *SOFA* Sequential Organ Failure Assessment score, *Lac-SOFA* sum of the SOFA score and the Lac-score.

The Kaplan–Meier survival curves showed that patients with higher SOFA class, Lac-SOFA score class, and Lac-scores had significantly worse 28-day survival rates (p < 0.05). When the patients were divided into nine subgroups according to both SOFA and Lac-SOFA score classes, the group of patients with Lac-scores sufficient to change the Lac-SOFA score class more than the SOFA score class had significantly worse 28-day survival rates than those with the same classification in both Lac-SOFA and SOFA scores (p < 0.05). Moreover, Kaplan–Meier survival curves were more differentiated by Lac-SOFA score class than by SOFA score class (Fig. 2). The number of patients in each of the subgroups is shown in Supplementary Fig. S7 online.

Logistic regression analysis for in-hospital mortality was performed with the entire study population for analysis of initial Lac-SOFA score (n = 6734). Age, BMI, history of several diseases (chronic lung disease, chronic neurological disease, hematologic malignancy, solid malignancy, and myocardial infarction), and initial Lac-SOFA score were identified as significant risk factors for in-hospital mortality in the multivariate logistic regression analysis (p < 0.05) (Supplementary Table S6 online).

The group whose difference of SOFA score between ICU days 3 and 1 was > 0 had an approximately four-fold higher risk of in-hospital mortality than the group whose difference of SOFA score between ICU days 3 and 1 was ≤ 0 (OR 3.91, CI 3.26–4.70). The group whose difference of Lac-SOFA score between ICU days 3 and 1 was > 0 had and approximately five-fold higher risk of in-hospital mortality than the group whose difference of Lac-SOFA score between ICU days 3 and 1 was ≤ 0 (OR 5.04, CI 4.16–6.11) (Supplementary Table S7 online).

In the sensitivity analysis of AUROCs for in-hospital mortality including only patients who had complete data, without imputation, (Supplementary Table S8 online) the findings were consistent with the findings for all patients. The ROC curves for in-hospital mortality based on the Delta Lac-score and lactate clearance between ICU days 1 and 3 (cutoffs − 50%, 0%, and 50%) are shown in Supplementary Fig. S8 online.

## Discussion

Accurate patient prognosis prediction is important because it can influence therapeutic decision making, guide resource allocation, and facilitate the provision of information or counseling by a physician to the patients and their relatives^1,17,18^. In this multicenter prospective cohort study, we evaluated a novel scoring system that was developed to predict mortality in patients with sepsis based on serial SOFA scores and serum lactate levels. Our results showed that the serial evaluation of lactate levels with the SOFA score, using the Lac-SOFA and Delta Lac-SOFA, may improve the predictive accuracy of the SOFA score for determining the mortality risk in patients with sepsis.

The SOFA score, which reflects the severity of organ dysfunction, has been widely used to predict the prognosis of patients with sepsis^3^. Many studies have been conducted to increase the accuracy of SOFA-based prognosis prediction in patients with sepsis. Ferreira et al. used serial SOFA scores, which were calculated at admission and every 48 h until discharge, and in univariate logistic regression, the mean SOFA score (odds ratios (OR), 3.06; 95% confidence intervals (CI), 2.36–3.97) and highest SOFA score (OR, 1.58; 95% CI, 1.43–1.76) had higher OR for mortality of critically ill patients than the initial SOFA score (OR, 1.45; 95% CI, 1.32–1.59), whereas the AUROC of the highest SOFA score (0.90; 95% CI, 0.86–0.93) was the highest among all of the assessed scores^1^. Ferreira et al.^1^ also reported that the AUROC of the SOFA score at 48 h (0.84; 95% CI, 0.78–0.88) was greater than that of the initial SOFA score (0.79; 95% CI, 0.75–0.83) for predicting mortality. In the present study, the AUROC of SOFA scores increased over time from initial (time zero of sepsis) to day 3 of ICU stay. This result indirectly supports the theory that early management of sepsis may improve patient outcomes^20^. Furthermore, we infer that the SOFA score of ICU days 2 or 3, which reflects organ failure after initial active treatment, better predicted mortality than the SOFA score of the time zero of sepsis or ICU day 1, which reflects organ failure at the beginning of sepsis. Moreover, the AUROC for predicting mortality reflected not only the progress of organ failure but also the improvement in the patient’s clinical condition after treatment. Therefore, the SOFA score or Lac-SOFA score after 24–48 h might be a more accurate predictor of in-hospital mortality than the initial scores.

Efforts to utilize serum lactate to predict the mortality of patients with sepsis have been undertaken previously. Yu et al.^21^ measured serial serum lactate levels at 2, 4, 6, and 12 h after septic shock. The lactate area score was defined as the area under the graph of serum lactate over time, which was calculated using the trapezoidal formula. The lactate area score (AUROC, 0.659; 95% CI, 0.597–0.720) better predicted 28-day mortality than the initial serum lactate level (AUROC, 0.596; 95% CI, 0.532–0.659). In multivariate logistic regression, only the lactate area score (OR, 1.013; 95% CI, 1.007–1.019) was significantly associated with 28-day mortality^21^. In the present study, the AUROC for in-hospital mortality of serum lactate on ICU day 1 was similar to the lactate area score of Yu et al. The AUROC for in-hospital mortality of serum lactate increased over time from the time zero of sepsis (initial) to day 3 of ICU stay. Furthermore, the abovementioned findings may be interpreted to infer that the serum lactate levels on ICU day 2 or 3 which reflect organ failure after initial active treatment better predicted mortality, which was similar to our results for the SOFA score.

Wright et al.^22^ showed that combining point-of-care lactate with the quick Sequential Organ Failure Assessment (qSOFA) score improved mortality prediction compared with the qSOFA score alone in patients admitted to hospital with suspected infection. However, to date, no studies have suggested a scoring system that uses both the SOFA score and the serum lactate level. In this study, the cutoff values of the Lac-score were set at 2, 4, 8, and 12 mmol/L. A lactate level of 4 mmol/L is known to be associated with severe sepsis^9,10,23^. Therefore, 8 and 12 mmol/L corresponding to 2 times and 3 times of 4 mmol/L were set as cutoff values for the Lac-score. Because all hospitals included in this study and other hospitals generally report an upper limit for serum lactate levels of ≥ 12 mmol/L, the Lac-score can be easily calculated and used in most circumstances. In the Sepsis-3 guidelines, blood lactate ≥ 2 mmol/L was newly added as one of the components of the definition of septic shock. Therefore, we set the Lac-score cutoff values to 2, 4, 8, and 12 mmol/L to form a score ranging from 0–4, similar to each item in the SOFA score. In-hospital mortality increased as the score of each component of the SOFA score increased, and this tendency was more evident with the Lac-score.

Our study showed that the Lac-SOFA score, which added Lac-score to the SOFA score, predicted in-hospital mortality more accurately than the SOFA score alone in patients with sepsis. This suggests that when added to the SOFA score, which evaluates the degree of hypoperfusion of major organs, serum lactate level, which reflects hypoperfusion status of the whole-body system, may function as a suitable parameter for predicting in-hospital mortality. The in-hospital mortality of ICU-treated sepsis was 41.9% according to results from a recent meta-analysis^24^. In the present study, the in-hospital mortality of patients with initial SOFA score ≤ 10 was less than 43.1%. In other words, patients with SOFA score of 10 or less in our study were expected to show similar in-hospital mortality when compared to a previous study. However, among patients with initial SOFA score ≤ 10, the in-hospital mortality of patients with initial Lac-SOFA of 12, 13, and 14 were 52.8%, 58.3%, and 75.0%, respectively. In patients with the same SOFA score, the addition of the Lac-score (i.e., the Lac-SOFA score) further differentiated in-hospital mortality among patients with sepsis. Moreover, our results showed that survival curves were more differentiated by Lac-SOFA score class than by SOFA score class. Therefore, using the Lac-SOFA score over the SOFA score alone may help better predict patients with a worse prognosis.

Initial active treatment is important in patients with sepsis^23^. However, as the existing scoring systems, such as SOFA, APACHE, and SAPS, are calculated from data recorded within 24 h, these systems do not reflect the patient's response to treatment and the changes in the clinical status^1^. Ryoo et al. predicted mortality in patients with septic shock according to serum lactate level and 6-h serum lactate clearance. The survivor group showed lower median 6-h lactate and higher 6-h lactate clearance than the non-survivor group. In logistic regression, the OR of lactate was 1.27 (95% CI, 1.21–1.34) and that of lactate clearance was 0.992 (95% CI, 0.989–0.995). The serum lactate level showed a higher predictive rate for mortality than serum lactate clearance (AUROC, 0.70 vs. 0.65; *p* < 0.01)^8^. In this study, we used the Delta Lac-score defined as the ratio of serum lactate on ICU day 3 to serum lactate on ICU day 1 (cutoff 0.5, 1, and 1.5) for evaluating progression of hypoperfusion over time, which is another expression of lactate clearance between ICU days 1 and 3 (cutoff − 50%, 0%, 50%; Lactate clearance between ICU days 1 and 3 [%] = [1 − Delta Lac-score] × 100 [%]). One previous study showed that 60-day mortality was significantly lower when the lactate clearance during 6 h increased by ≥ 10%^25^. Another study showed that in patients with severe sepsis or septic shock, the change in lactate clearance during 6 h was 13% ± 38% in survivors and − 13% ± 7% in non-survivors, and the change in lactate clearance during 24 h was 42% ± 33% in survivors and − 17% ± 76% in non-survivors^26^. Converting this result to the lactate ratio, the lactate level after 24 h was 0.58 times the initial lactate level in survivors and 1.17 times the initial lactate level in non-survivors. When imputation was performed for 48 h, the lactate level was 0.34 times and 1.37 times the initial lactate level in survivors and non-survivors, respectively. Therefore, in the present study, the cutoffs value of the Delta Lac-score were set at 0.5, 1, and 1.5. When comparing the in-hospital mortality AUROCs for the Delta Lac-score and that of lactate clearance between ICU days 1 and 3 (cutoffs − 50%, 0%, and 50%), the two AUROCs for in-hospital mortality were exactly the same. We inferred that the serum lactate level reflects disease severity on the day of measurement, and the Delta Lac-score contains different types of information on the degree of improvement of the disease over time. Therefore, by using these two different types of data, the Delta Lac-SOFA score better reflects disease severity, as shown by the highest AUROC for in-hospital mortality.

The current study has important clinical implications. The Lac-SOFA score is significantly better than the SOFA score at predicting in-hospital mortality. The survival curves were more differentiated by the Lac-SOFA score class than by the SOFA score class, indicating that the Lac-SOFA score may be more accurate than the SOFA score in predicting the mortality risk in patients with sepsis. The in-hospital mortality AUROCs for Lac-SOFA score and SOFA score on initial and ICU day 1 had moderate power (0.65–0.73). However, the in-hospital mortality AUROCs for Lac-SOFA score and SOFA score increased significantly from ICU day 1 to ICU day 3. Patients whose Lac-SOFA score increased between ICU days 1 and 3 was > 0 had a five-fold higher risk of in-hospital mortality than those whose Lac-SOFA score remained unchanged or decreased between ICU days 1 and 3. In addition, the Delta Lac-SOFA showed the highest discrimination power in the prediction of in-hospital mortality. Accordingly, our findings show that combining serial evaluation of lactate levels with the SOFA score may improve the predictive accuracy of the SOFA score for determining the mortality risk in patients with sepsis. Furthermore, the Lac-SOFA score at the time zero of sepsis or on ICU day 1, which reflects organ failure at the beginning of sepsis, can be used to determine the initial treatment strategy more appropriately, while the Lac-SOFA score on ICU day 2 or 3, which reflects the progress of organ failure, can be used to assess the treatment response. Therefore, during the early course of ICU treatment, this novel scoring system may reflect the patient's response to treatment and the changes in clinical status, and have an important role by influencing therapeutic decision-making, guiding resource allocation, and facilitating information-sharing or counseling of patients with sepsis and their relatives by physicians.

This study has several limitations. First, our study compared ICU days 1 to 3 serial SOFA scores and Lac-SOFA scores between the early-ICU and delayed ICU groups; thus, patients who died or were discharged from the ICU within 3 days were not included in this study. The imputation was performed using data from the initial measurements to ICU day 3 to reduce immortal time or information bias. However, a sensitivity analysis showed consistent findings when the analysis included only patients who had complete data without imputation. Second, this study used sepsis registry data collected from 19 tertiary hospitals in Korea. As this was not a multinational study, it is not possible to evaluate the generalizability of these results to other countries, and this warrants further studies. Third, the SOFA score was originally named as the “Sepsis-Related” Organ Failure Assessment score, but because this score was applicable to critically ill patients without sepsis, the name was changed to “Sequential” Organ Failure Assessment^11^. However, this study was limited to patients with sepsis, in whom the lactate level is known to play an important role. Therefore, additional research is needed to apply this new scoring system to critically ill patients with conditions other than sepsis.

In conclusion, serial evaluation of lactate levels with the SOFA score, by using the Lac-SOFA and Delta Lac-SOFA, may improve the utility of the SOFA score for predicting in-hospital mortality in patients with sepsis. This novel scoring system reflects the patient’s response to early treatment and the changes in the clinical status, enabling better clinical decision-making and resource allocation.

## Materials and methods

### Study design and subjects

This study was a secondary analysis of a nationwide, multicenter, prospective, observational cohort study of patients with sepsis that was conducted between September 2019 and December 2020. Data on hospital-onset sepsis and community-onset sepsis were obtained from a sepsis registry created by the Korean Sepsis Alliance, which consists of 19 tertiary or university-affiliated hospitals in South Korea that agreed to participate in the study. This study was performed in line with the principles of the Declaration of Helsinki. The Institutional Review Board of Seoul National University hospital approved this study (IRB-H-1808-135-967). As this was an observational study, the decision to obtain or waive written informed consent was left to the discretion of the institutional review boards of the participating hospitals. It was confirmed that all procedures adhered to the relevant guidelines and regulations. The present study conformed to the STROBE Statement.

Adult patients (age ≥ 19 years) who were diagnosed with sepsis were included and followed up until discharge from the hospital or death. Sepsis was diagnosed according to the Sepsis-3 definition^3^. The missing baseline SOFA score was assumed to be zero. Exclusion criteria included patients with initial SOFA score < 2 and patients without initial serum lactate results and without available serum lactate results on day 1 of ICU.

### Diagnosis of sepsis in the sepsis registry

In the case of community-onset sepsis diagnosed in the emergency room, the screening criteria were met when all of the following conditions were satisfied: patient’s age 19 years or higher at the time of admission to the emergency room, satisfying at least two of the three qSOFA criteria in their first-recorded vital signs at the time of admission to the emergency room (respiration ≥ 22 cycles/min, systolic blood pressure ≤ 100 mmHg, loss of consciousness; if the assessment of the level of consciousness is impossible, one or more of the other two conditions should be satisfied). In the case of hospital-onset sepsis diagnosed in the ward, the screening criteria were met when all of the following conditions were satisfied: case of sepsis, which was identified by the rapid response team; patient's age 19 years or higher at the time of recognition of sepsis. In the case of community-onset sepsis, the time of the emergency room triage visit was defined as time zero. In the case of hospital-onset sepsis, time zero was defined as the time-point when the patient was first diagnosed with sepsis by the rapid response team. The worst test result was used in patients in whom the same test was performed several times within a day in the ICU. Based on the vital signs and laboratory results performed within 1 h from time zero, if both of the following criteria were satisfied, the patient was diagnosed with sepsis, and only patients who met the sepsis diagnostic criteria were included in the study: if infection was suspected or confirmed; the SOFA score increased by more than 2 points compared to the baseline SOFA score.

### Study outcomes and variables

The main outcome was in-hospital mortality. Data collected from the sepsis registry included: interval from time zero to ICU admission, interval from time zero to hospital discharge; age, sex, BMI, and comorbidities as baseline characteristics; the initial SOFA score, SOFA score on ICU days 1, 2, and 3; the initial serum lactate, serum lactate on ICU days 1, 2, and 3 using arterial blood; and in-hospital mortality. For the initial data, the result closest to time zero of sepsis was used.

### Statistical analysis

Fisher's exact test and Pearson’s chi-square test were used to compare categorical variables. The Mann–Whitney *U* test, Kruskal–Wallis test, and Student's *t*-test were used to analyze continuous variables. The results were considered significant with two-tailed *p*-values less than 0.05. If there was a single missing data for the serum lactate level or SOFA score, the imputation was conducted to obtain the average value using the sequential data^1^. If there were no data available for initial (time zero of sepsis) or ICU day 1, the available ICU day 1 or initial data were used. The time interval for available data that can be replaced with each other was set within 2 h from time zero^27^. We used the AUROC analysis to evaluate the prediction of mortality, and applied DeLong's test to compare two correlated ROC curves. The Kaplan–Meier method with log-rank test was used to estimate survival probability and the patients discharged alive prior to 28 days were presumed alive at 28 days. Logistic regression was used to analyze the risk factors that affect mortality, including independent variables which are known risk factors for mortality in patients with sepsis, and variables with *p* < 0.1 in the univariate analysis were included in the multivariate logistic regression analysis^28–32^. The results are presented as OR with 95% CI. The Statistical Package for the Social Sciences (SPSS; version 22.0; IBM corp., Armonk, NY, USA) was used for analysis.

## Supplementary Information

Supplementary Information.

## Data Availability

The datasets generated during the current study are available from the corresponding author on request.

## References

[1] Ferreira FL, Bota DP, Bross A, Mélot C, Vincent JL (2001). Serial evaluation of the SOFA score to predict outcome in critically ill patients. JAMA.

[2] Vincent J-L (1998). Use of the SOFA score to assess the incidence of organ dysfunction/failure in intensive care units: Results of a multicenter, prospective study. Crit. Care Med..

[3] Singer M (2016). The third international consensus definitions for sepsis and septic shock (Sepsis-3). JAMA.

[4] Oh Y (2020). Sequential Organ Failure Assessment score as a predictor of mortality in ventilated patients with multidrug-resistant bacteremia. Acute Crit. Care.

[5] Casserly B (2015). Lactate measurements in sepsis-induced tissue hypoperfusion: Results from the Surviving Sepsis Campaign database. Crit. Care Med..

[6] Jansen TC (2010). Early lactate-guided therapy in intensive care unit patients: A multicenter, open-label, randomized controlled trial. Am. J. Respir. Crit. Care Med..

[7] Puskarich MA (2013). Whole blood lactate kinetics in patients undergoing quantitative resuscitation for severe sepsis and septic shock. Chest.

[8] Ryoo SM (2018). Lactate level versus lactate clearance for predicting mortality in patients with septic shock defined by Sepsis-3. Crit. Care Med..

[9] BouChebl R (2017). Serum lactate is an independent predictor of hospital mortality in critically ill patients in the emergency department: A retrospective study. Scand. J. Trauma Resusc. Emerg. Med..

[10] Gotmaker R, Peake SL, Forbes A, Bellomo R, ARISE Investigators (2017). Mortality is greater in septic patients with hyperlactatemia than with refractory hypotension. Shock.

[11] Moreno R (2023). The Sequential Organ Failure Assessment (SOFA) Score: Has the time come for an update?. Crit. Care.

[12] Bion JF, Aitchison TC, Edlin SA, Ledingham IM (1988). Sickness scoring and response to treatment as predictors of outcome from critical illness. Intensive Care Med..

[13] Larvin M, McMahon MJ (1989). APACHE-II score for assessment and monitoring of acute pancreatitis. Lancet.

[14] Sawyer R (1992). Peritonitis into the 1990s: Changing pathogens and changing strategies in the critically ill. Am. Surg..

[15] Moreno R (1999). The use of maximum SOFA score to quantify organ dysfunction/failure in intensive care. Results of a prospective, multicentre study. Working Group on Sepsis related Problems of the ESICM. Intensive Care Med..

[16] Goldhill DR, Sumner A (1998). Outcome of intensive care patients in a group of British intensive care units. Crit. Care Med..

[17] Cullen DJ, Chernow B (1994). Predicting outcome in critically ill patients. Crit. Care Med..

[18] Shortell SM (1994). The performance of intensive care units: Does good management make a difference?. Med. Care.

[19] Evans L (2021). Surviving Sepsis Campaign: International guidelines for management of sepsis and septic shock 2021. Crit. Care Med..

[20] Dellinger RP (2013). Surviving Sepsis Campaign: International guidelines for management of severe sepsis and septic shock, 2012. Intensive Care Med..

[21] Yu G (2019). Utility of the early lactate area score as a prognostic marker for septic shock patients in the emergency department. Acute Crit. Care.

[22] Wright SW (2022). Enhanced bedside mortality prediction combining point-of-care lactate and the quick Sequential Organ Failure Assessment (qSOFA) score in patients hospitalised with suspected infection in southeast Asia: A cohort study. Lancet Glob. Health.

[23] Levy MM, Evans LE, Rhodes A (2018). The Surviving Sepsis Campaign Bundle: 2018 update. Crit. Care Med..

[24] Fleischmann-Struzek C (2020). Incidence and mortality of hospital- and ICU-treated sepsis: Results from an updated and expanded systematic review and meta-analysis. Intensive Care Med..

[25] Nguyen HB (2004). Early lactate clearance is associated with improved outcome in severe sepsis and septic shock. Crit. Care Med..

[26] Marty P (2013). Lactate clearance for death prediction in severe sepsis or septic shock patients during the first 24 hours in Intensive Care Unit: An observational study. Ann. Intensive Care.

[27] Vincent J-L, Quintairos E Silva A, Couto L, Taccone FS (2016). The value of blood lactate kinetics in critically ill patients: A systematic review. Crit. Care.

[28] Martin GS, Mannino DM, Moss M (2006). The effect of age on the development and outcome of adult sepsis. Crit. Care Med..

[29] Yang Y, Yang KS, Hsann YM, Lim V, Ong BC (2010). The effect of comorbidity and age on hospital mortality and length of stay in patients with sepsis. J. Crit. Care.

[30] Maizel J (2013). Impact of non-dialysis chronic kidney disease on survival in patients with septic shock. BMC Nephrol..

[31] Lemay AC, Anzueto A, Restrepo MI, Mortensen EM (2014). Predictors of long-term mortality after severe sepsis in the elderly. Am. J. Med. Sci..

[32] López-Mestanza C, Andaluz-Ojeda D, Gómez-López JR, Bermejo-Martín JF (2018). Clinical factors influencing mortality risk in hospital-acquired sepsis. J. Hosp. Infect..

