# Supplementary material for: Serial evaluation of the serum lactate level with the SOFA score to predict mortality in patients with sepsis
Source: Sci Rep. 2023 Apr 18;13:6351. doi: 10.1038/s41598-023-33227-7 (PMC10113181; doi:10.1038/s41598-023-33227-7)
Supplement: Supplementary file 1 — Supplementary Information. [file 41598_2023_33227_MOESM1_ESM.pdf]

Serial evaluation of the serum lactate level with the SOFA score to predict mortality in patients with sepsis

Authors:

Heemoon Park, MD[1]; Jinwoo Lee, MD[1]; Dong Kyu Oh, MD[2]; Mi Hyeon Park[2]; Chae-Man Lim, MD, PhD[2]; Sang-Min Lee, MD, PhD[1,3]; *Hong Yeul Lee, MD[3]; on behalf of the Korean Sepsis Alliance (KSA) investigators

Affiliations:

[1]Division of Pulmonary and Critical Care Medicine, Department of Internal Medicine, Seoul National University hospital, Seoul National University College of Medicine, Seoul, Republic of Korea

[2]Department of Pulmonary and Critical Care Medicine, Asan Medical Center, University of Ulsan College of Medicine, Seoul, Republic of Korea

[3]Department of Critical Care Medicine, Seoul National University hospital, Seoul, Republic of Korea

Corresponding author:

*Hong Yeul Lee, M.D.

Assistant Professor

Department of Critical Care Medicine, Seoul National University hospital, 101 Daehak-ro, Jongno-gu, Seoul, 03080, Republic of Korea.

Tel: 82-2-2072-2957

Fax: 82-2-762-9662

E-mail: takumama@naver.com

Supplementary Figure Legends

Supplementary Fig. S1 Flow chart of the study participant selection

ICU, intensive care unit; SOFA, Sequential Organ Failure Assessment score; Lac-SOFA, sum of the SOFA score and the Lac-score; Delta Lac-SOFA, sum of the Lac-SOFA ICU day-3 and Delta Lac-scores

Supplementary Fig. S2 Receiver-operating characteristic (ROC) curves for in-hospital mortality based on the SOFA and Lac-SOFA scores

(a) ROC curves for in-hospital mortality based on the initial SOFA and initial Lac-SOFA scores in the delayed-ICU group; (b) ROC curves for in-hospital mortality based on the initial SOFA and initial Lac-SOFA scores in the non-ICU group; (c) ROC curves for in-hospital mortality based on the initial SOFA and initial Lac-SOFA scores in the early-ICU group; (d) ROC curves for in-hospital mortality based on the SOFA and Lac-SOFA scores on ICU day 1 in the early-ICU group; (e) ROC curves for in-hospital mortality based on the SOFA and Lac-SOFA scores on ICU day 2 in the early-ICU group; (f) ROC curves for in-hospital mortality based on the SOFA and Lac-SOFA scores on ICU day 3 in the early-ICU group;

ICU, intensive care unit; SOFA, Sequential Organ Failure Assessment score; Lac-SOFA, sum of the SOFA score and the Lac-score

Supplementary Fig. S3 Receiver-operating characteristic (ROC) curves for in-hospital mortality based on the SOFA and Lac-SOFA scores on ICU day 1, 2, and 3 in delayed-ICU group

(a) ROC curves for in-hospital mortality based on the SOFA and Lac-SOFA scores on ICU day 1 in the delayed-ICU group; (b) ROC curves for in-hospital mortality based on the SOFA and Lac-SOFA scores on ICU day 2 in the delayed-ICU group; (c) ROC curves for in-hospital mortality based on the SOFA and Lac-SOFA scores on ICU day 3 in the delayed-ICU group

ICU, intensive care unit; SOFA, Sequential Organ Failure Assessment score; Lac-SOFA, sum of the SOFA score and the Lac-score

Supplementary Fig. S4 Receiver-operating characteristic (ROC) curves for in-hospital mortality based on the SOFA and Lac-SOFA on ICU day 3, and Delta Lac-SOFA scores

(a) ROC curves for in-hospital mortality based on the SOFA on ICU day 3 and Delta Lac-SOFA scores in the

early-ICU group; (b) ROC curves for in-hospital mortality based on the SOFA on ICU day 3 and Delta Lac-SOFA scores in the delayed-ICU group; (c) ROC curves for in-hospital mortality based on the Lac-SOFA on ICU day 3 and Delta Lac-SOFA scores in the early-ICU group; (d) ROC curves for in-hospital mortality based on the Lac-SOFA on ICU day 3 and Delta Lac-SOFA scores in the delayed-ICU group

ICU, intensive care unit; SOFA, Sequential Organ Failure Assessment score; Lac-SOFA, sum of the SOFA score and the Lac-score; Delta Lac-SOFA, sum of the Lac-SOFA ICU day-3 and Delta Lac-scores

Supplementary Fig. S5 In-hospital mortality according to the score of each component of the SOFA score and the Lac-score

SOFA, Sequential Organ Failure Assessment

Supplementary Fig. S6 Difference in-hospital mortality according to the initial Lac-score in patients with the same initial SOFA score

As the Lac-score ranged from 0 to 4, each patient's initial SOFA score was multiplied by 5 and added to the patient's initial Lac-score. Therefore, in-hospital mortality was compared between patients with the same SOFA score but different Lac-scores.

SOFA, Sequential Organ Failure Assessment

Supplementary Fig. S7 Number of patients according to the changes in the initial Lac-SOFA or Delta Lac-SOFA scores

Patients were divided into five or six subgroups with 5-point intervals of the (a) initial Lac-SOFA or (b) Delta Lac-SOFA scores. The numbers above the bar show the number of patients.

ICU, intensive care unit; SOFA, Sequential Organ Failure Assessment score; Lac-SOFA, sum of the SOFA score and the Lac-score; Delta Lac-SOFA, sum of the Lac-SOFA ICU day-3 and Delta Lac-scores

Supplementary Fig. S8 Receiver-operating characteristic (ROC) curves for in-hospital mortality based on the Delta Lac-score and lactate clearance between ICU days 1 and 3 (cutoffs −50%, 0%, and 50%)

ICU, intensive care unit

Supplementary Table Legends

Supplementary Table S1 In-hospital mortality according to Lac-scores with or without Delta Lac-score in the non-ICU, early-ICU, and delayed-ICU groups

Supplementary Table S2 AUROCs for in-hospital mortality based on the serial SOFA score, serial serum lactate levels, serial Lac-scores, and Delta Lac-score in the early-ICU and delayed-ICU groups

Supplementary Table S3 Difference in the compliance with the hour-1 sepsis bundle according to the class of the Delta Lac-SOFA score (by 5-point intervals)

Supplementary Table S4 AUROCs for in-hospital mortality based on the serial SOFA score, lactate level, Lac-score, Delta Lac-score and the Lac-score ICU day 3 plus Delta Lac-score in the all participants

Supplementary Table S5 In-hospital mortality according to initial Lac-score, SOFA score and Lac-SOFA score in the entire study population for analysis of initial Lac-SOFA score with initial SOFA score ≤10

Supplementary Table S6 Logistic regression analysis of the entire study population to assess parameters associated with in-hospital mortality

Supplementary Table S7 In-hospital mortality according to the difference in the SOFA or Lac-SOFA score between ICU days 3 and 1

Supplementary Table S8 AUROCs for in-hospital mortality based on the serial SOFA score, Lac-SOFA score, and Delta Lac-SOFA score in patients with complete data (N=2859)

Supplementary Figures

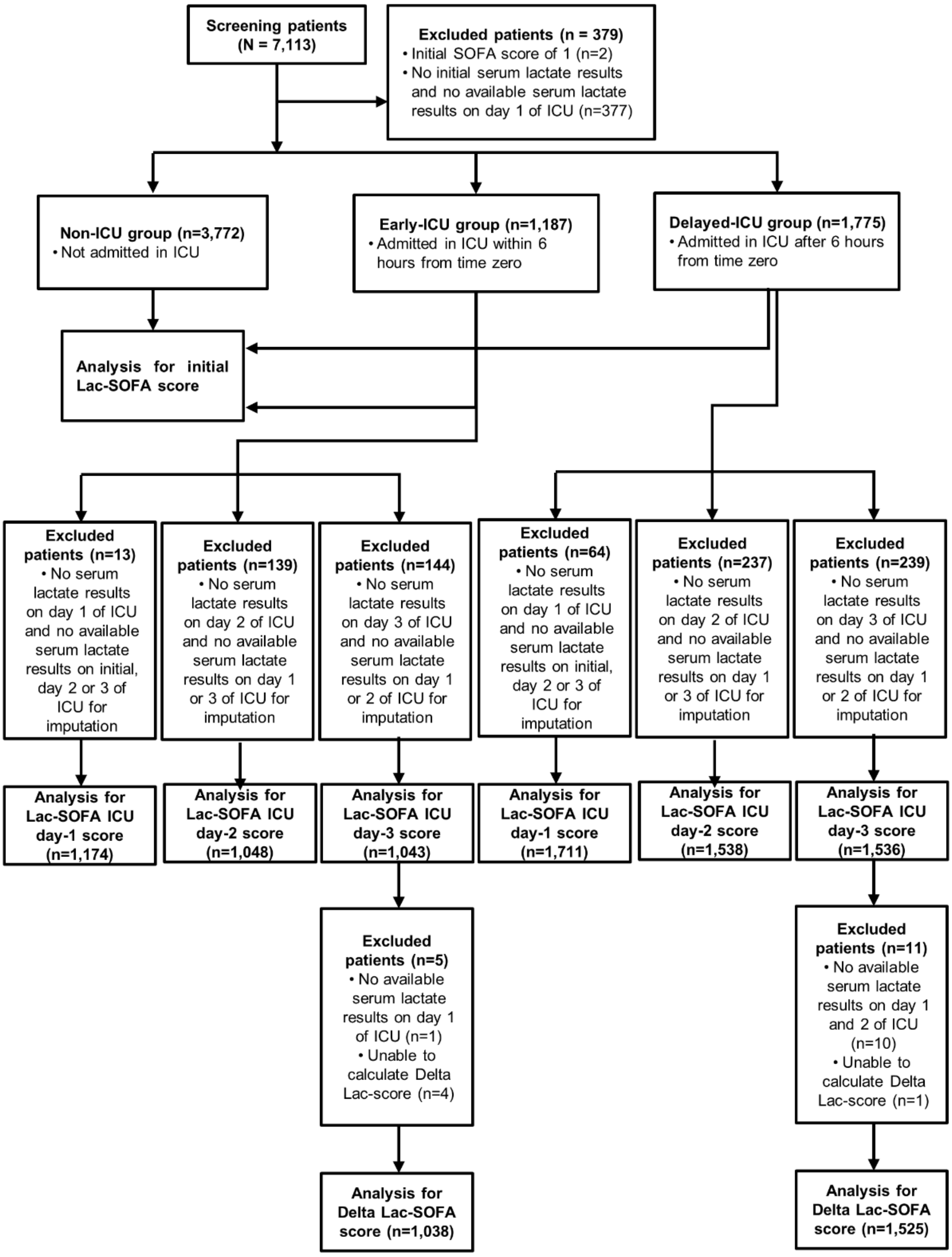

Supplementary Fig. S1 Flow chart of the study participant selection

ICU, intensive care unit; SOFA, Sequential Organ Failure Assessment score; Lac-SOFA, sum of the SOFA score and the Lac-score; Delta Lac-SOFA, sum of the Lac-SOFA ICU day-3 and Delta Lac-scores

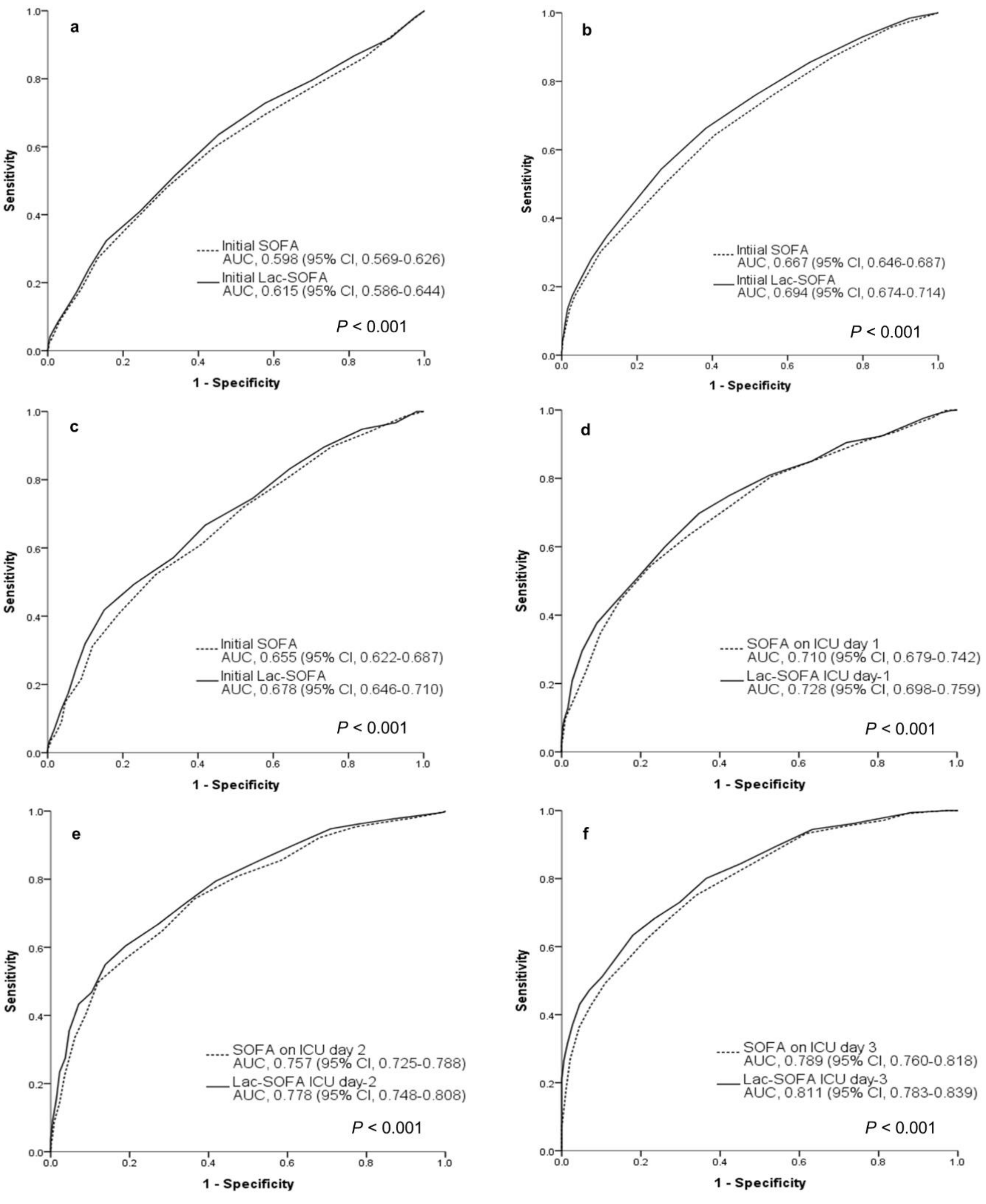

Supplementary Fig. S2 Receiver-operating characteristic (ROC) curves for in-hospital mortality based on the SOFA and Lac-SOFA scores

(a) ROC curves for in-hospital mortality based on the initial SOFA and initial Lac-SOFA scores in the delayed-ICU group; (b) ROC curves for in-hospital mortality based on the initial SOFA and initial Lac-SOFA scores in the non-ICU group; (c) ROC curves for in-hospital mortality based on the initial SOFA and initial Lac-SOFA scores in the early-ICU group; (d) ROC curves for in-hospital mortality based on the SOFA and Lac-SOFA scores on

ICU day 1 in the early-ICU group; (e) ROC curves for in-hospital mortality based on the SOFA and Lac-SOFA scores on ICU day 2 in the early-ICU group; (f) ROC curves for in-hospital mortality based on the SOFA and Lac-SOFA scores on ICU day 3 in the early-ICU group;

ICU, intensive care unit; SOFA, Sequential Organ Failure Assessment score; Lac-SOFA, sum of the SOFA score and the Lac-score

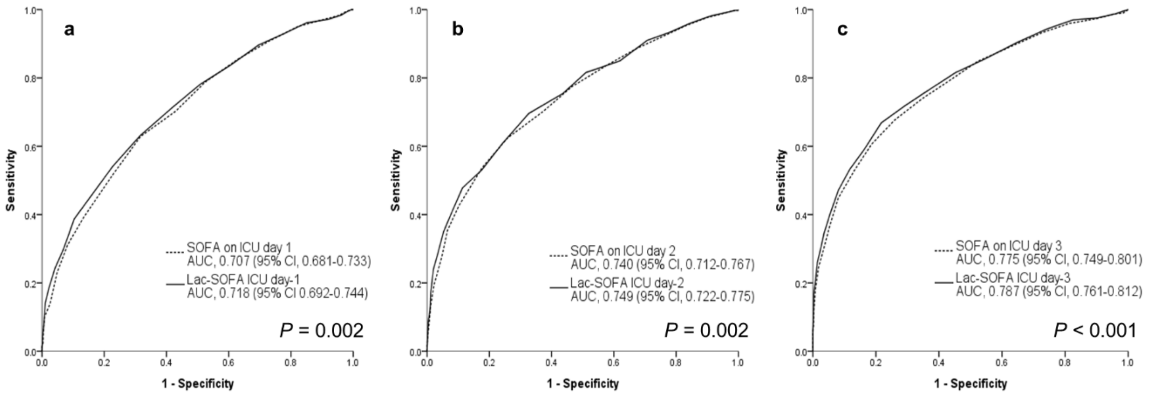

Supplementary Fig. S3 Receiver-operating characteristic (ROC) curves for in-hospital mortality based on the SOFA and Lac-SOFA scores on ICU day 1, 2, and 3 in delayed-ICU group

(a) ROC curves for in-hospital mortality based on the SOFA and Lac-SOFA scores on ICU day 1 in the delayed-ICU group; (b) ROC curves for in-hospital mortality based on the SOFA and Lac-SOFA scores on ICU day 2 in the delayed-ICU group; (c) ROC curves for in-hospital mortality based on the SOFA and Lac-SOFA scores on ICU day 3 in the delayed-ICU group

ICU, intensive care unit; SOFA, Sequential Organ Failure Assessment score; Lac-SOFA, sum of the SOFA score and the Lac-score

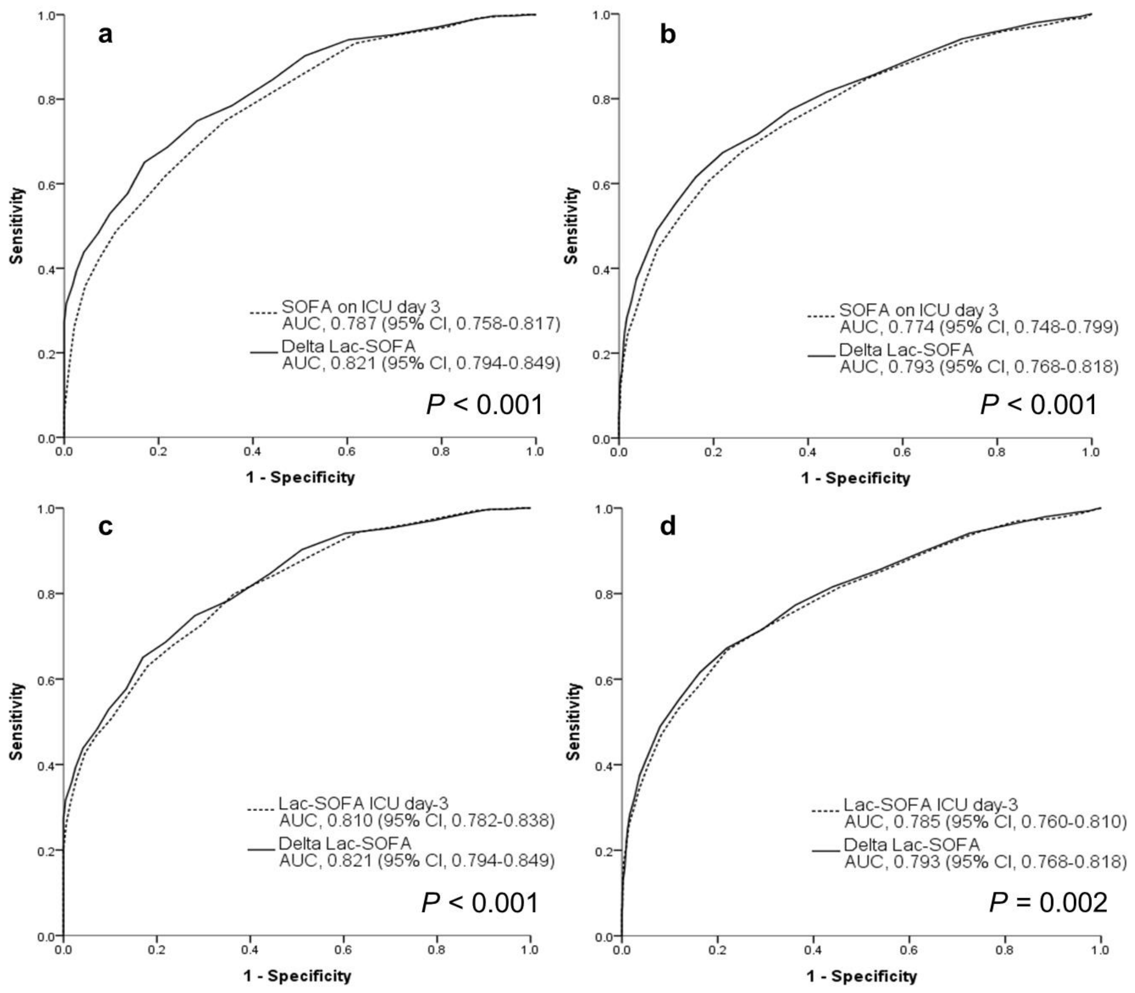

Supplementary Fig. S4 Receiver-operating characteristic (ROC) curves for in-hospital mortality based on the SOFA and Lac-SOFA on ICU day 3, and Delta Lac-SOFA scores

(a) ROC curves for in-hospital mortality based on the SOFA on ICU day 3 and Delta Lac-SOFA scores in the early-ICU group; (b) ROC curves for in-hospital mortality based on the SOFA on ICU day 3 and Delta Lac-SOFA scores in the delayed-ICU group; (c) ROC curves for in-hospital mortality based on the Lac-SOFA on ICU day 3 and Delta Lac-SOFA scores in the early-ICU group; (d) ROC curves for in-hospital mortality based on the Lac-SOFA on ICU day 3 and Delta Lac-SOFA scores in the delayed-ICU group

ICU, intensive care unit; SOFA, Sequential Organ Failure Assessment score; Lac-SOFA, sum of the SOFA score and the Lac-score; Delta Lac-SOFA, sum of the Lac-SOFA ICU day-3 and Delta Lac-scores

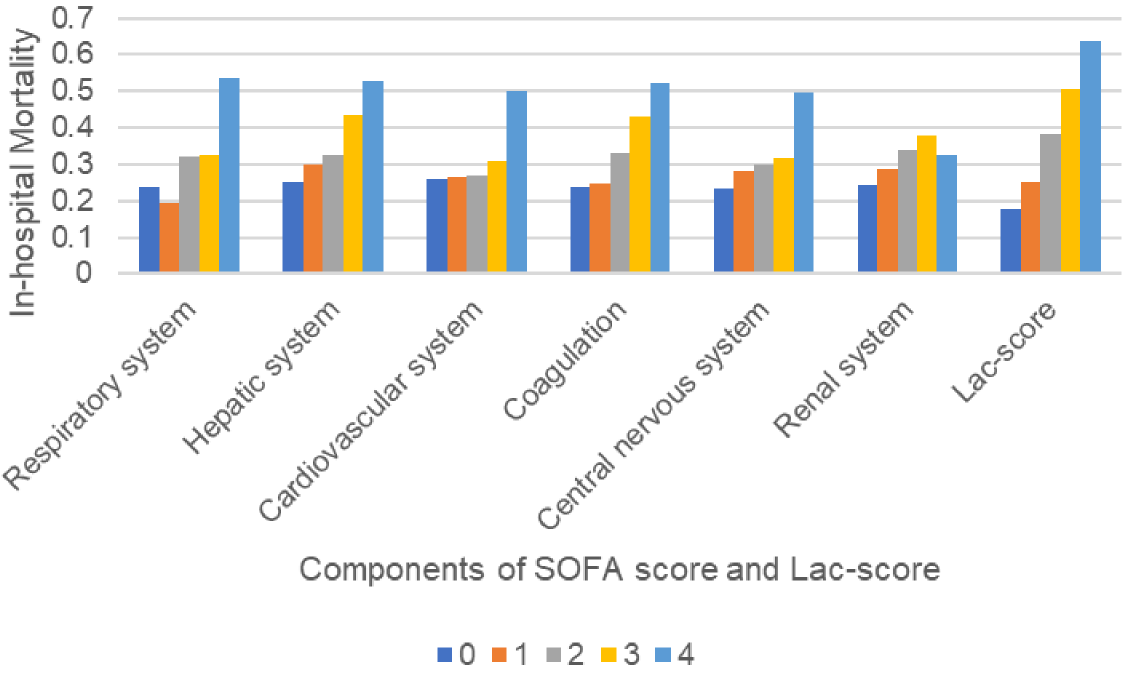

Supplementary Fig. S5 In-hospital mortality according to the score of each component of the SOFA score and the Lac-score

SOFA, Sequential Organ Failure Assessment

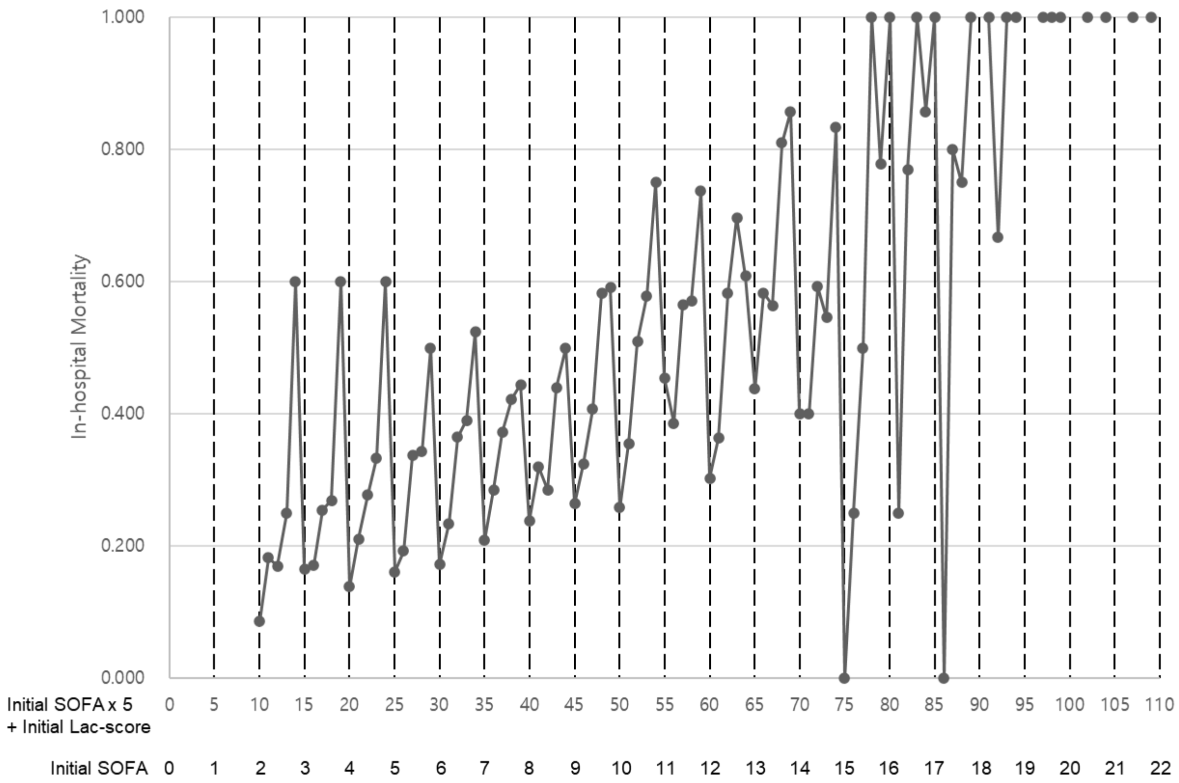

Supplementary Fig. S6 Difference in-hospital mortality according to the initial Lac-score in patients with the same initial SOFA score

As the Lac-score ranged from 0 to 4, each patient's initial SOFA score was multiplied by 5 and added to the patient's initial Lac-score. Therefore, in-hospital mortality was compared between patients with the same SOFA score but different Lac-scores.

SOFA, Sequential Organ Failure Assessment

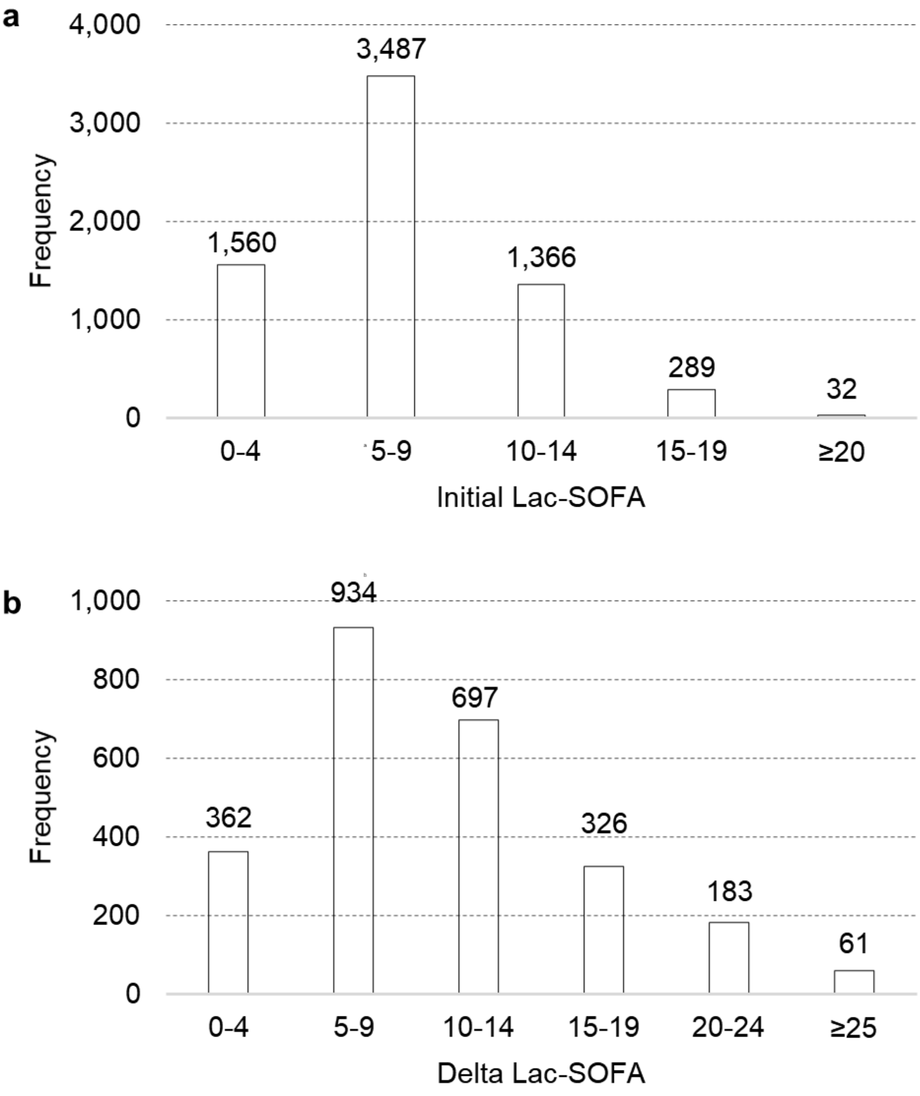

Supplementary Fig. S7 Number of patients according to the changes in the initial Lac-SOFA or Delta Lac-SOFA scores

Patients were divided into five or six subgroups with 5-point intervals of the (a) initial Lac-SOFA or (b) Delta Lac-SOFA scores. The numbers above the bar show the number of patients.

ICU, intensive care unit; SOFA, Sequential Organ Failure Assessment score; Lac-SOFA, sum of the SOFA score and the Lac-score; Delta Lac-SOFA, sum of the Lac-SOFA ICU day-3 and Delta Lac-scores

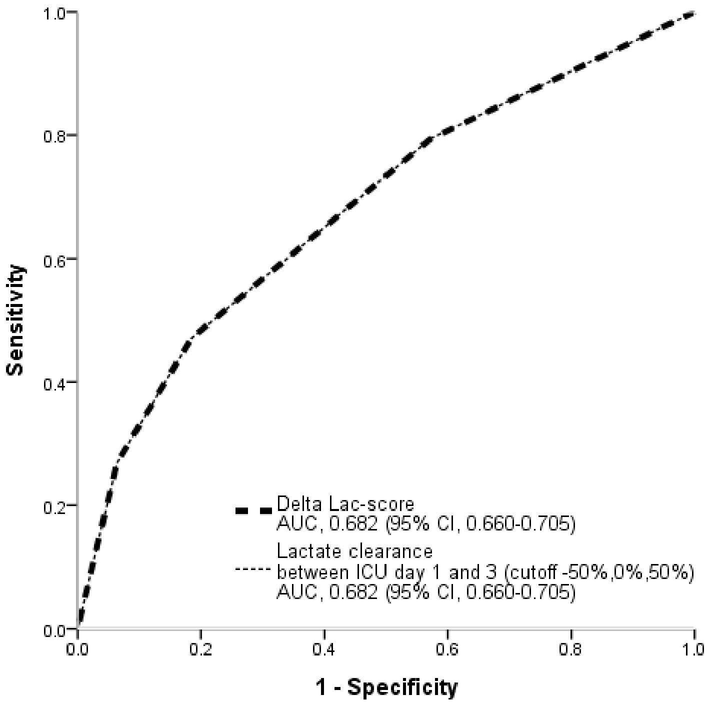

Supplementary Fig. S8 Receiver-operating characteristic (ROC) curves for in-hospital mortality based on the Delta Lac-score and lactate clearance between ICU days 1 and 3 (cutoffs −50%, 0%, and 50%)

ICU, intensive care unit

Supplementary Tables

Supplementary Table S1 In-hospital mortality according to Lac-scores with or without Delta Lac-score in the non-ICU, early-ICU, and delayed-ICU groups

Variable			0	1	2	3	4	5	6	7
In-hospital mortality, n/N (%)	Lac-score, initial	Non-ICU	219/1545 (14.2%)	179/1273 (21.9%)	243/675 (36.0%)	93/181 (51.4%)	67/98 (68.4%)			
		Early-ICU	74/328 (22.6%)	93/328 (28.4%)	150/340 (44.1%)	52/110 (47.3%)	54/81 (66.7%)			
		Delayed-ICU	136/541 (25.1%)	172/573 (30.0%)	178/477 (37.3%)	68/130 (52.3%)	28/54 (51.9%)			
	Lac-score ICU day-1	Early-ICU	78/357 (21.8%)	94/345 (27.2%)	112/270 (41.5%)	51/92 (55.4%)	85/110 (77.3%)			
		Delayed-ICU	145/645 (22.5%)	147/514 (28.6%)	150/352 (42.6%)	72/123 (58.5%)	57/77 (74.0%)			
	Lac-score ICU day-2	Early-ICU	75/479 (15.7%)	80/271 (29.5%)	69/143 (48.3%)	46/68 (67.6%)	76/87 (87.4%)			
		Delayed-ICU	154/746 (20.6%)	126/426 (29.6%)	99/208 (47.6%)	45/73 (61.6%)	76/85 (89.4%)			
	Lac-score ICU day-3	Early-ICU	95/618 (15.4%)	75/218 (34.4%)	57/87 (65.5%)	25/28 (89.3%)	89/92 (96.7%)			
		Delayed-ICU	179/944 (19.0%)	119/328 (36.3%)	76/129 (58.9%)	39/51 (76.5%)	82/84 (97.6%)			
	Lac-score ICU day-3 plus Delta Lac-score	Early-ICU	34/306 (11.1%)	68/319 (21.3%)	44/149 (29.5%)	47/89 (52.8%)	30/49 (61.2%)	28/36 (77.8%)	30/31 (96.8%)	57/59 (96.6%)
		Delayed-ICU	64/405 (15.8%)	106/461 (23.0%)	88/271 (32.5%)	54/143 (37.8%)	45/87 (51.7%)	42/59 (71.2%)	37/42 (88.1%)	57/57 (100%)

ICU, intensive care unit

Supplementary Table S2 AUROCs for in-hospital mortality based on the serial SOFA score, serial serum lactate levels, serial Lac-scores, and Delta Lac-score in the early-ICU and delayed-ICU groups.

Variable	Early-ICU group		Delayed-ICU group	
	AUROC	*p*-value	AUROC	*p*-value
SOFA, initial	0.655±0.017	†<0.001	0.598±0.015	†<0.001
SOFA on ICU day 1	0.710±0.016	†<0.001	0.707±0.013	†<0.001
SOFA on ICU day 2	0.757±0.016	†<0.001	0.740±0.014	†<0.001
SOFA on ICU day 3	0.789±0.015	†<0.001	0.775±0.013	†<0.001
Lactate, initial	0.650±0.017	†<0.001	0.595±0.015	†<0.001
Lactate on ICU day 1	0.692±0.016	†<0.001	0.668±0.014	†<0.001
Lactate on ICU day 2	0.769±0.016	†<0.001	0.713±0.014	†<0.001
Lactate on ICU day 3	0.810±0.015	†<0.001	0.760±0.014	†<0.001
Lac-score, initial	0.644±0.017	†<0.001	0.593±0.014	†<0.001
Lac-score ICU day-1	0.683±0.017	†<0.001	0.653±0.014	†<0.001
Lac-score ICU day-2	0.753±0.017	†<0.001	0.688±0.015	†<0.001
Lac-score ICU day-3	0.785±0.017	†<0.001	0.725±0.015	†<0.001
Delta Lac-score	0.714±0.018	†<0.001	0.660±0.015	†<0.001
Lac-score ICU day-3 plus Delta Lac-score	0.795±0.016	†<0.001	0.726±0.015	†<0.001

ROC, receiver-operating characteristic; AUROC, area under the ROC curve; SOFA, sequential organ failure assessment score; ICU, intensive care unit; †*p*-value < 0.05

Supplementary Table S3 Difference in the compliance with the hour-1 sepsis bundle according to the class of the Delta Lac-SOFA score (by 5-point intervals)

Variables, n (%)		Class of the Delta Lac-SOFA score						*p*-value
		1	2	3	4	5	6	
Sepsis bundle within 1h	Measure lactate level	281 (77.6)	773 (82.8)	551 (79.1)	263 (80.7)	147 (80.3)	52 (85.2)	0.238
	Obtain blood cultures	247 (68.2)	627 (67.1)	441 (63.3)	205 (62.9)	122 (66.7)	41 (67.2)	0.418
	Administer antibiotics	108 (29.8)	251 (26.9)	207 (29.7)	89 (27.3)	47 (25.7)	11 (18.0)	0.332
	Fluid resuscitation	153 (75.0)	396 (74.9)	313 (71.5)	173 (75.9)	113 (72.9)	41 (74.5)	0.809
	Start vasopressors	101 (49.5)	257 (48.6)	221 (50.5)	110 (48.2)	85 (54.8)	24 (43.6)	0.700

Supplementary Table S4 AUROCs for in-hospital mortality based on the serial SOFA score, lactate level, Lac-score, Delta Lac-score and the Lac-score ICU day 3 plus Delta Lac-score in the all participants

Variable (A)	AUROC (95% CI)	Variable (B)	AUROC (95% CI)	*p*-value [a)]
SOFA, initial	0.656 (0.641-0.670)	Lactate, initial	0.657 (0.642-0.672)	0.867
SOFA, initial	0.656 (0.641-0.670)	Lac-score, initial	0.650 (0.635-0.664)	0.491
Lactate, initial	0.657 (0.642-0.672)	Lac-score, initial	0.650 (0.635-0.664)	†<0.001
SOFA on ICU day 1	0.709 (0.689-0.729)	Lactate on ICU day 1	0.678 (0.657-0.699)	†0.006
SOFA on ICU day 1	0.709 (0.689-0.729)	Lac-score ICU day-1	0.666 (0.645-0.686)	†<0.001
Lactate on ICU day 1	0.678 (0.657-0.699)	Lac-score ICU day-1	0.666 (0.645-0.686)	†<0.001
SOFA on ICU day 2	0.747 (0.726-0.767)	Lactate on ICU day 2	0.736 (0.715-0.757)	0.306
SOFA on ICU day 2	0.747 (0.726-0.767)	Lac-score ICU day-2	0.715(0.694-0.736)	†0.003
Lactate on ICU day 2	0.736 (0.715-0.757)	Lac-score ICU day-2	0.715(0.694-0.736)	†<0.001
SOFA on ICU day 3	0.779 (0.760-0.798)	Lactate on ICU day 3	0.779 (0.759-0.800)	0.967
SOFA on ICU day 3	0.779 (0.760-0.798)	Lac-score ICU day-3	0.749 (0.729-0.768)	†0.003
SOFA on ICU day 3	0.779 (0.760-0.798)	Delta Lac-score	0.682 (0.661-0.704)	†<0.001
SOFA on ICU day 3	0.779 (0.760-0.798	Lac-score ICU day-3 plus Delta Lac-score	0.755 (0.734-0.775)	†0.031
Lactate on ICU day 3	0.779 (0.759-0.800)	Lac-score ICU day-3	0.749 (0.729-0.768)	†<0.001
Lactate on ICU day 3	0.779 (0.759-0.800)	Delta Lac-score	0.682 (0.661-0.704)	†<0.001
Lactate on ICU day 3	0.779 (0.759-0.800)	Lac-score ICU day-3 plus Delta Lac-score	0.755 (0.734-0.775)	†<0.001
Lac-score ICU day-3	0.749 (0.729-0.768)	Delta Lac-score	0.682 (0.661-0.704)	†<0.001
Lac-score ICU day-3	0.749 (0.729-0.768)	Lac-score ICU day-3 plus Delta Lac-score	0.755 (0.734-0.775)	0.366
Delta Lac-score	0.682 (0.661-0.704)	Lac-score ICU day-3 plus Delta Lac-score	0.755 (0.734-0.775)	†<0.001

ROC, receiver-operating characteristic; AUROC, area under the ROC curve; SOFA, sequential organ failure assessment score; ICU, intensive care unit; CI, confidence interval; [a)] two-tailed DeLong's test for two correlated ROC curves (A) and (B); †*p*-value < 0.05

Supplementary Table S5 In-hospital mortality according to initial Lac-score, SOFA score and Lac-SOFA score in the entire study population for analysis of initial Lac-SOFA score with initial SOFA score ≤10

	In-hospital mortality, n/N (%)		
Score	Lac-score, initial	SOFA, initial	Lac-SOFA, initial
0	391/2319 (16.9%)	‥	‥
1	486/2029 (24.0%)	‥	‥
2	428/1253 (34.2%)	74/545(13.6%)	25/288 (8.7%)
3	133/309 (43.0%)	146/774 (18.9%)	100/584 (17.1%)
4	77/140 (55.0%)	164/848 (19.3%)	105/688 (15.3%)
5	‥	198/902 (22.0%)	151/779 (19.4%)
6	‥	221/855 (25.8%)	152/743 (20.5%)
7	‥	217/731 (29.7%)	199/764 (26.0%)
8	‥	186/619 (30.0%)	202/670 (30.1%)
9	‥	168/449 (37.4%)	178/531 (33.5%)
10	‥	141/327 (43.1%)	144/451 (31.9%)
11	‥	‥	119/300 (39.7%)
12	‥	‥	93/176 (52.8%)
13	‥	‥	35/60 (58.3%)
14	‥	‥	12/16 (75.0%)

ICU, intensive care unit; SOFA, sequential organ failure assessment score; Lac-SOFA score, sum of SOFA score and Lac-score

Supplementary Table S6 Logistic regression analysis of the entire study population to assess parameters associated with in-hospital mortality

Variable	Univariable analysis	Multivariable analysis
	Odds ratio (95% CI)	Odds ratio (95% CI)
Age	†1.007 [1.003, 1.011]	†1.012 [1.007, 1.017]
Sex	†0.852 [0.764, 0.949]	
Body mass index	†0.980 [0.967, 0.993]	†0.969 [0.955, 0.983]
History of chronic lung disease	†1.204 [1.045, 1.387]	†1.291 [1.099, 1.516]
History of chronic neurological disease	†0.724 [0.636, 0.824]	†0.803 [0.689, 0.935]
History of chronic liver disease	†1.220 [1.028, 1.447]	
History of chronic kidney disease	1.152 [0.992, 1.339]	
History of hematologic malignancy	†1.970 [1.624, 2.390]	†2.221 [1.786, 2.763]
History of solid malignancy	†1.519 [1.362, 1.693]	†1.808 [1.593, 2.053]
History of chronic heart failure	†1.313 [1.079, 1.597]	
History of myocardial infarction	†1.151 [0.959, 1.382]	1.205 [0.984, 1.475]
History of vascular disease	†0.811 [0.710, 0.927]	
Lac-SOFA, initial	†1.206 [1.187, 1.225]	†1.209 [1.189, 1.229]

ICU, intensive care unit; SOFA, sequential organ failure assessment score; Lac-SOFA score, sum of SOFA score and Lac-score; CI, confidence interval; †p-value<0.05

Supplementary Table S7 In-hospital mortality according to the difference in the SOFA or Lac-SOFA score between ICU days 3 and 1

	Difference in score between ICU days 3 and 1 ≤0		Difference in score between ICU days 3 and 1 > 0		
Variable	Total	Mortality, n (%)	Total	Mortality, n (%)	Odds ratio (95% CI)
SOFA score	1856	442 (23.8%)	707	389 (55.0%)	3.91 (3.26–4.70)
Lac-SOFA score	1937	452 (23.3%)	626	379 (60.5%)	5.04 (4.16–6.11)

Lac-SOFA score, sum of SOFA score and Lac-score; ICU, intensive care unit; CI, confidence interval; SOFA, Sequential Organ Failure Assessment; * p-value<0.05

Supplementary Table S8 AUROCs for in-hospital mortality based on the serial SOFA score, Lac-SOFA score, and Delta Lac-SOFA score in patients with complete data (N=2859)

Variable (A)	AUROC (95% CI)	Variable (B)	AUROC (95% CI)	*p*-value [a)]
SOFA on ICU day 2	0.706 (0.681-0.730)	SOFA on ICU day 1	0.656 (0.630-0.682)	†<0.001
SOFA on ICU day 3	0.751 (0.729-0.774)	SOFA on ICU day 2	0.706 (0.681-0.730)	†<0.001
Lac-SOFA ICU day-1	0.722 (0.702-0.742)	SOFA on ICU day 1	0.708 (0.688-0.728)	†<0.001
Lac-SOFA ICU day-2	0.759 (0.738-0.779)	SOFA on ICU day 2	0.745 (0.724-0.765)	†<0.001
Lac-SOFA ICU day-3	0.767 (0.745-0.789)	SOFA on ICU day 3	0.753 (0.731-0.776)	†<0.001
Delta Lac-SOFA	0.777 (0.755-0.798)	SOFA on ICU day 3	0.751 (0.729-0.774)	†<0.001
Delta Lac-SOFA	0.777 (0.755-0.798)	Lac-SOFA ICU day-3	0.765 (0.743-0.788)	†<0.001

ROC, receiver-operating characteristic; AUROC, area under the ROC curve; SOFA, sequential organ failure assessment score; Lac-SOFA score, sum of SOFA score and Lac-score; ICU, intensive care unit; CI, confidence interval; [a)] two-tailed DeLong's test for two correlated ROC curves (A) and (B); †*p*-value < 0.05
